# A melatonin textile transdermal patch based on physicochemical synergistic permeation-enhancement strategy for circadian rhythm regulation

**DOI:** 10.3389/fbioe.2026.1913854

**Published:** 2026-10-08

**Authors:** Juyang Zhao, Jinzhi Liu, Jiahan Lin, Mingtao Liu, Fujun Wang, Lu Wang, Jing Gao

**Affiliations:** 1 Shanghai Frontiers Science Center of Advanced Textiles, College of Textiles, Donghua University, Shanghai, China; 2 Key Laboratory of Textile Industry for Biomedical Textile Materials and Technology, Donghua University, Shanghai, China

**Keywords:** electret, insomnia, melatonin, permeation enhancer, transdermal delivery

## Abstract

Melatonin, an endogenous circadian rhythm-regulating hormone, has faced challenges in achieving prolonged exogenous regulation due to its low oral bioavailability and short half-life. Existing transdermal strategies are difficult to balance efficacy, safety, and accessibility. To address this, we propose a physicochemical synergistic permeation-enhancement strategy and have developed a melatonin textile transdermal patch based on the synergistic action of electret and permeation enhancer. The patch is designed with a three-layer composite structure, composed of a textile-based electret backing layer, a drug-loading pressure sensitive adhesive layer containing melatonin and permeation enhancer, and a protective layer to prevent drug leakage. Electret and permeation enhancer enhance transdermal efficiency through a physicochemical synergistic permeation-enhancement strategy without disrupting the skin barrier. *In vitro* studies revealed that among various permeation enhancers (menthol, azone, oleic acid) and electret parameters, the combination of a −1500 V electret with 10% oleic acid exhibited the optimal synergistic effect. *In vivo* pharmacokinetic studies demonstrated that the patch reached a peak plasma concentration (>30 ng/mL) within 1 h and maintained effective therapeutic levels (>5 ng/mL) for up to 8 h. The patch significantly improved the sleep quality in insomniac rats, with no observed skin irritation or cytotoxicity. This study not only accomplishes an efficient, non-invasive, and parametrically tunable transdermal delivery of melatonin but also validates the feasibility of synergistic enhancement through physical field regulation and chemical permeation, thereby providing a viable strategy for developing safe and long-acting textile-based transdermal drug delivery systems aimed at neuroendocrine small-molecule hormones similar to melatonin.

## 1 Introduction

The human circadian rhythm meticulously regulates key physiological processes such as the sleep-wake cycle, metabolism, immune function, and neuroendocrine activities (Hartley et al., 2018; Focke et al., 2020). However, in modern society, lifestyle changes, increased mental stress, and environmental factors such as nocturnal light pollution and blue light exposure from electronic devices have highlighted the issue of circadian rhythm disruption (Gentry et al., 2021; Rao et al., 2024). Studies indicate that circadian rhythm disruption affects up to one-third of the global adult population (Perlis et al., 2022). It not only significantly impairs sleep quality and daytime function but has also been identified as an independent risk factor for several major diseases. It is closely associated with mental disorders such as anxiety and depression, and markedly increases the risk of metabolic syndrome, cardiovascular diseases, neurodegenerative disorders, and even malignant tumors, posing a growing global health challenge. The resulting public health burden and economic losses are staggering (Fishbein et al., 2021; Fatima et al., 2020; Walker et al., 2020; Schrader et al., 2024).

Clinical studies have confirmed that patients with circadian rhythm disorders exhibit reduced nocturnal melatonin secretion. Exogenous melatonin supplementation has been demonstrated to significantly restore circadian homeostasis and shows definitive efficacy in managing insomnia (Slomski et al., 2018; Jafari-Koulaee et al., 2021; Auld et al., 2017). Melatonin (N-acetyl-5-methoxytryptamine (Lerchl et al., 2012)) is an endogenous hormone synthesized primarily by the pineal gland within the human brain (Gandhi et al., 2015). As a key regulator of the circadian rhythm (Megha et al., 2024; Valdés-Tovar et al., 2018), melatonin binds to G-protein-coupled receptors, upregulates the levels of γ-aminobutyric acid (GABA) synthesizing enzymes in the brain (Dubocovich et al., 2010), and transmits sleep signals to the central nervous system, thereby reducing sleep latency and extending sleep duration (Wang et al., 2024).

Currently, exogenous melatonin supplementation is primarily achieved *via* oral administration in clinical practice. However, its application is severely limited by melatonin’s short half-life (0.35–0.50 h *in vivo*) (Cardinali et al., 2012), low oral bioavailability (approximately 15%) (Harpsøe et al., 2015), potential for gastrointestinal irritation, and the additional metabolic burden it places on the liver and kidneys. Transdermal drug delivery, which can bypass first-pass metabolism and maintain steady-state plasma drug concentrations, is considered an ideal alternative (Jiang et al., 2020; He et al., 2023). In recent years, various strategies have been explored to enhance the transdermal efficiency of melatonin, including microneedles (e.g., silk fibroin microneedles), nanoparticles (e.g., nanovesicles), and chemical permeation enhancers (Li et al., 2025; Qi et al., 2021; Kandimalla et al., 2016; Jana et al., 2017; Kim et al., 2021). However, these permeation-enhancement strategies possess inherent limitations: (1) While microneedles can penetrate the stratum corneum, they suffer from low drug-loading capacity and carry risks of micro-injuries and infection (Dul et al., 2023); (2) Although chemical permeation enhancers (e.g., azone, menthol) can efficiently facilitate drug diffusion within the stratum corneum by altering skin barrier properties, excessive concentrations of these enhancers often cause skin irritation or allergic reactions, and may even induce irreversible or long-term damage to the skin barrier function (Chen et al., 2016; Li et al., 2024; Tang et al., 2023; Nagai et al., 2019); (3) Nanocarriers, despite their sustained-release potential, involve complex preparation processes, high costs, and unresolved long-term biosafety concerns (Zhou et al., 2018). Therefore, developing an efficient, non-invasive, safe, and readily scalable method for melatonin delivery remains crucial (Comai et al., 2024).

In recent years, significant progress has been made in stimuli-responsive transdermal delivery platforms, providing new avenues to overcome the limitations of conventional penetration enhancement strategies. Shah et al. (2025) systematically reviewed the advantages, challenges, and emerging applications of innovative transdermal drug delivery systems. For instance, a self-heating hierarchical microneedle patch has been developed for efficient local treatment of skin cancer *via* thermally enhanced drug release (Shao et al., 2024). Additionally, a thermally enhanced self-heating microneedle patch has also been applied for the sustained delivery of progesterone, demonstrating the clinical translation potential of thermal-responsive strategies in transdermal drug delivery (Shen et al., 2026). These studies indicate that strategies combining physical stimuli (such as heat or electric fields) with microneedle or patch technologies are emerging as important directions for improving transdermal delivery efficiency. In contrast to the above-mentioned studies that rely on microneedles or complex thermal-responsive materials, our study is based on a physicochemical synergistic penetration enhancement strategy, utilizing the combined action of electret and chemical penetration enhancer to develop a structurally simplified textile-based transdermal patch, aiming to provide a novel solution for the non-invasive and long-acting delivery of melatonin.

Electrets represent a typical, mild, and non-invasive physical permeation-enhancement technology, referring to dielectric materials capable of long-term retention of space charges and dipole charges (Jeong et al., 2021; Tu et al., 2016). When applied to the skin, an electret can modulate the arrangement of stratum corneum lipids *via* its surface electrostatic field, thereby reversibly enhancing the transdermal permeability of various drugs. However, relying solely on an electret may not achieve sufficient permeation flux or plasma concentration to produce a stable therapeutic effect (Jiang et al., 2008). Studies have shown that the combined use of an electret and a chemical permeation enhancer, after parameter optimization, can produce a synergistic enhancement of transdermal delivery for certain drugs, offsetting the limited efficiency of an electret alone while lowering the enhancer concentration needed to reduce irritation risk (Cui et al., 2012; Cui et al., 2013). However, the electret materials employed in existing studies are mostly films, which fail to effectively dissipate water vapor evaporated from the skin, leading to its accumulation at the application interface. This not only causes a stuffy, moist, and uncomfortable sensation on the skin but also may degrade or even disable the electrical performance of the electret due to excessive local humidity (Viraneva et al., 2010). More importantly, no study has yet reported on the synergistic enhancement of transdermal melatonin delivery by combining an electret with a chemical permeation enhancer, nor has there been development of a transdermal patch system that integrates both strategies while ensuring both delivery efficacy and wearing comfort.

Consequently, this study aims to address the current lack of research on the synergistic enhancement of transdermal melatonin delivery by combining an electret with a chemical permeation enhancer. In addition, targeting the key limitations of existing film-based electrets, namely, poor breathability, susceptibility to electrical performance degradation due to moisture accumulation, and wearing discomfort, we have developed a melatonin textile transdermal patch based on a physicochemical synergistic permeation-enhancement strategy (Scheme 1). This drug-in-adhesive (DIA) system (Galge et al., 2022) consists of a breathable textile-based electret backing layer, a pressure-sensitive adhesive (PSA) drug reservoir containing melatonin and a permeation enhancer, and a protective liner to prevent drug leakage. Upon application to the skin, melatonin and the permeation enhancer in the PSA passively diffuse to the skin surface. The permeation enhancer and the electret simultaneously act on the stratum corneum *via* chemical and physical mechanisms, respectively, synergistically disrupting the skin barrier and thereby effectively enhancing the transdermal delivery of melatonin. Notably, the textile-based electret, by virtue of its excellent breathable structure, not only significantly improves wearing comfort but also effectively prevents local moisture accumulation that could otherwise impair the electrical performance of the electret, thus helping to maintain its long-lasting permeation-enhancing effect. We systematically optimized the textile-based electret parameters as well as the type and concentration of the permeation enhancer. The delivery efficacy was comprehensively evaluated using an *in vitro* transdermal diffusion model and *in vivo* pharmacokinetic studies in insomniac rats, confirming that the patch shortens sleep latency, prolongs sleep duration, and offers comfort, safety, and long-acting efficacy. This work provides a new strategy for transdermal melatonin delivery that is mechanism-driven, operationally straightforward, comfortable, long-acting, and holds promising potential for clinical translation.

**SCHEME 1:**
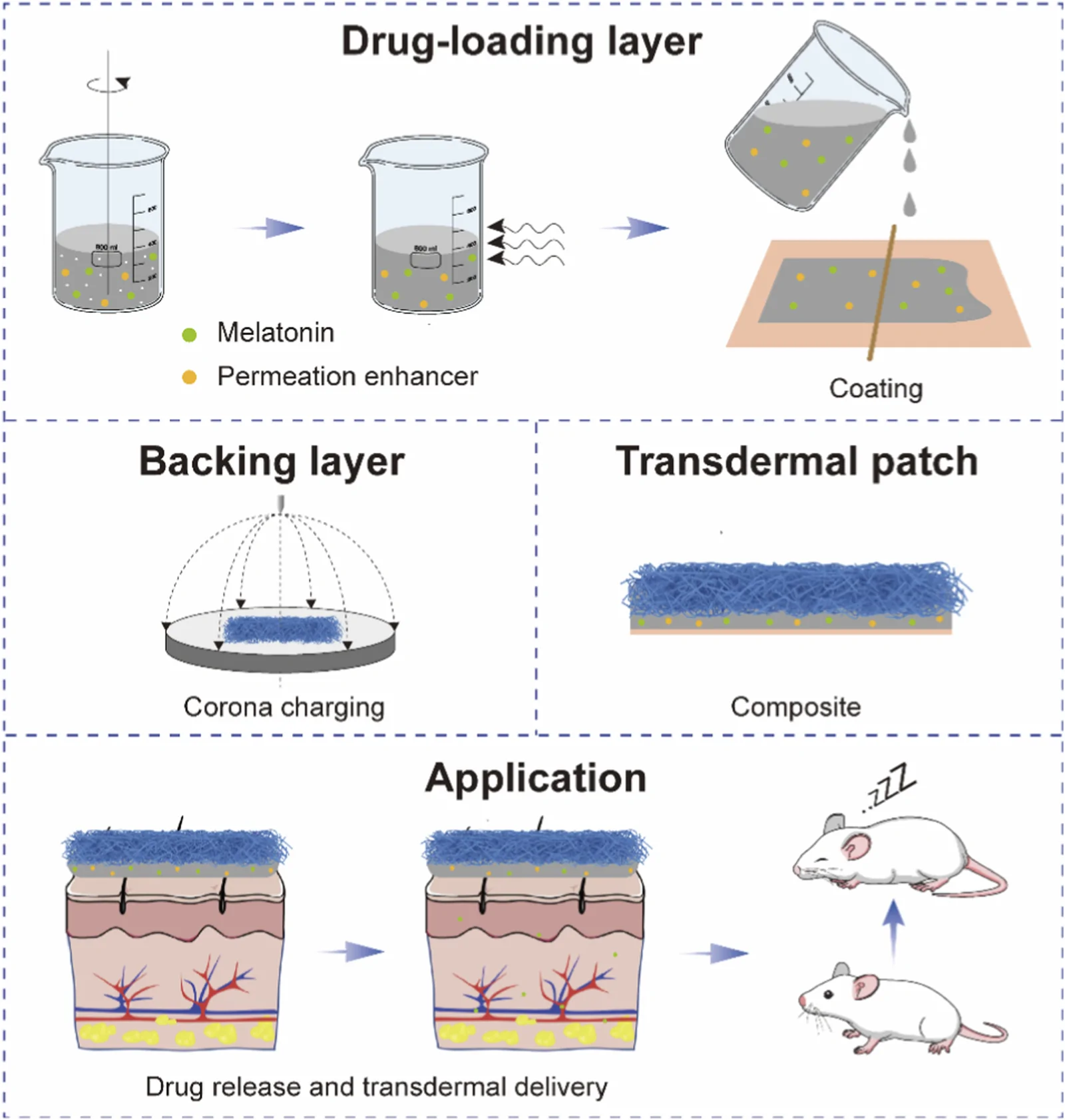
The design strategy, synthetic route and application of the electret-permeation enhancer synergetic melatonin textile transdermal patch.

## 2 Materials and methods

### 2.1 Materials

Melatonin and oleic acid were purchased from Aladdin (Shanghai, China). Polyacrylate pressure-sensitive adhesive (Duro-Tak 387-2510) was purchased from Loctite (Sinzing, Germany). Menthol was purchased from Sigma-Aldrich (St. Louis, Missouri). Azone, ethyl acetate, methanol and potassium bromide (KBr) were purchased from Sinopharm (Shanghai, China). Polypropylene nonwoven fabric was purchased from Tiantianxiangshang (Taizhou, China). Glassine paper was purchased from Qiongxiu (Shenzhen, China). Laboratory mice (C57) and rats (SD) were provided by Donghua University Animal Center (Shanghai, China).

### 2.2 Preparation of electret-permeation enhancer synergetic transdermal patch of melatonin

The drug-loaded layer of transdermal patch was prepared using a solvent casting technique. A 20% melatonin (MT) solution in ethyl acetate was incorporated into the PSA at a fixed MT-to-PSA mass ratio of 1:47, with permeation enhancers added according to specific experimental formulations. The mixture was magnetically stirred at 200 rpm for 2 h, followed by 15-min ultrasonication for defoaming. The mixture was evenly coated on the Glassine paper using the MS-ZN320B coating machine. The semi-finished product was left static at room temperature for 15 min, and then placed in a vacuum drying oven at 37 °C for 20 min.

The polypropylene (PP) non-woven fabric was charged for 10 min by a gate-controlled constant voltage corona charging system, and the gate voltages were set to± 500 V, ±1000 V, and ±1500 V, respectively.

Finally, the PP nonwoven fabric was laminated as the backing layer on the drug-loading layer, and the prepared patch was cut into 3 × 3 cm, 2 × 2 cm and 1 × 1 cm squares for further evaluation.

The drug-loaded control patches were fabricated using an uncharged PP nonwoven fabric and containing no permeation enhancer.

### 2.3 Scanning electron microscopy (SEM)

The patch was frozen in liquid nitrogen for 30 s and then cut with a scalpel rapidly. The sample was mounted on an aluminum stub using conductive carbon tape and sputter-coated with a 20-nm gold layer under vacuum. The characterization of the cross-section morphology of the patch was carried out through scanning electron microscopy, using a Hitachi FlexSEM 1,000 scanning microscope.

### 2.4 Adhesion of transdermal patch

Adhesion properties were evaluated in accordance with the Chinese Pharmacopoeia (ChP 2020) methodology. The rolling ball test was used to evaluate the tack of patches, and the time required for patches to completely disengage from the experimental plate under a fixed tensile force was measured to evaluate the holding-power of patches. All samples were measured in triplicate.

### 2.5 Air permeability of transdermal patch

Using a fabric air permeability meter, a constant 200 Pa pressure difference is maintained on both sides of the sample with an area of 20 cm^2^. The air permeability of the samples (n = 3) can be calculated by testing the air flow rate per unit area of the sample through the sample per unit time. Before the test, each sample was balanced in a standard atmospheric pressure environment with a temperature of 20 °C ± 2 °C and a relative humidity of 65% ± 2%.

### 2.6 Distribution coefficient of backing layer

PP nonwovens of 40, 60, 80 g/m^2^ were cut into 1 × 1 cm squares (n = 3), and respectively added to a centrifuge tube containing 4 mL of 20 μg/mL melatonin solution, where the solvent was 1:49 (v/v) methanol and water. The centrifuge tube was sealed and placed in a shaker at 37 °C for 24 h, then was centrifuged at 10,000 rpm for 20 min to take the supernatant. Then the content of melatonin in the supernatant was analyzed by ultraviolet spectrophotometry. According to the concentration of melatonin in the solution before and after equilibrium, the distribution coefficient was calculated by Equation 1.

$$k=\frac{\left(C_{0}-C\right)}{C}$$ (1)

where C_0_ and C represent the concentration of melatonin in the solution before and after distribution, respectively.

### 2.7 Fourier transform infrared spectroscopy (FTIR)

FTIR was performed by Nicolet 6,700 infrared spectrometer. PP nonwovens were divided into two groups. One group was immersed in 4 mL of 20 μg/mL melatonin solution, where the solvent was 1: 49 (v/v) methanol and water. The other group was immersed in 4 mL of the same solvent. Both were placed in a shaker at 37 °C for 24 h and then thoroughly dried in an oven. The dried samples were co-ground with KBr to prepare the sample. Subsequently, the changes in absorption peak values within the scanning range of 400–4,000 cm^-1^ are tested using a FTIR.

### 2.8 Preparation of isolated skin samples

The skin of C57 mice was used as a skin model. After shaving, the back skin of the mice was peeled off, and the subcutaneous tissue was removed. The peeled mouse skin was unfolded on a glass plate, and the skin surface was gently pressed with tape, and then the stratum corneum on the surface of the tape was peeled together, repeated for about 25 times until the stratum corneum of the skin was completely peeled off to obtain viable skin.

### 2.9 *In vitro* drug release

The self-made *in vitro* release test device was used for testing. The experimental device is mainly composed of a stirring paddle, a dissolution cup, a mesh disc and a water bath system. The dissolution cup is a bottom hemispherical glass container, and the mesh disc is a stainless-steel mesh with a wire diameter of 0.9 mm, and the diameter is the same as that of the dissolution cup. The patch was fixed on the mesh disc. 500 mL of phosphate buffer solution with pH 6.5 was placed in the dissolution cup, which was heated in a water bath at 32 °C, and the stirring paddle speed was 100 rpm. 1 mL of dissolution medium was extracted at 0.5, 1, 2, 4, 6 and 8 h, respectively, and the same volume of blank medium was supplemented. The content of melatonin in dissolution medium was determined by ultraviolet spectrophotometry. Each experiment was performed in triplicate. The drug loading of melatonin (MT) in each patch sample is 6 mg.

### 2.10 *In vitro* transdermal permeation study

Using the Franz diffusion cell test, the isolated skin was placed at the mouth of the diffusion pool and subjected to liquid phase cutting with the normal saline in the pool. After the protective layer of the patch was uncovered, it was closely attached to the center of the isolated skin. The whole device was kept at 32 °C. At 0.5, 1, 2, 4, 6 and 8 h, all the receiving solutions in the diffusion cell were sucked, and the same volume of blank receiving solution was supplemented. The content of melatonin in the receiving solution was determined by ultraviolet spectrophotometry. Each experiment was performed in triplicate. The drug loading of melatonin (MT) in each patch sample is 6 mg.

### 2.11 Biocompatibility testing

Preparation of patch extracts. The patches were cut into pieces with a surface area of 1 cm^2^, sterilized with ethylene oxide, and placed in centrifuge tubes. Complete medium was added at a ratio of 1 cm^2^ per mL, followed by incubation in a culture incubator for 10 h.

Cytotoxicity assay. The cytotoxicity of the patch was evaluated using a CCK-8 kit and a Live/Dead Double Staining Kit. HaCaT cells were cultured with the patch extract, and cell viability in the extract was measured at 450 nm using a microplate reader after 24 h. Following 24-h culture in the patch extract, HaCaT cells were stained with the Live/Dead Double Staining Kit (Beyotime, Shanghai, China) and observed under a fluorescence microscope (Ex/Em = 490/515 nm for Calcein-AM and Ex/Em = 535/617 nm for PI). Each experiment was performed in triplicate.

### 2.12 *In vivo* transdermal permeation study

An insomnia rat model was established. Male SD rats weighing 200–250 g were selected and randomized into normal and model groups for subsequent experiments. The model was induced by intraperitoneal injection of a PCPA suspension (40 mg/mL), which was prepared in a weakly alkaline saline solution (composed of NaHCO_3_ and NaCl). The suspension was administered at a dose of 400 mg/kg for three consecutive days.

Following successful model establishment, the control group (n = 3) received no treatment. All the remaining rats were anesthetized with 5% isoflurane (RWD Life Science, Shenzhen, China) inhalation for 5 min for induction, followed by maintenance under 3% isoflurane during the treatment. For the drug-loaded control patch group (n = 3), the hair on a specific area of the back was shaved. After removing the protective liner, the 2 × 2 cm drug-loaded control patch was applied firmly to the skin. The rats of the drug-loaded transdermal patch group (n = 3) were treated with the 2 × 2 cm drug-loaded transdermal patches using the same procedure. All rats had free access to food and water throughout the experimental period. Plasma melatonin levels were measured, and sleep-like behaviors were observed before the experiment and at 0.5, 1, 2, 4, 6, 8, and 10 h after patch application.

Blood samples were collected from the rats *via* tail tipping at different time points after patch administration. Specifically, 0.1 mL of blood was drawn and placed into tubes containing EDTA-2Na as an anticoagulant. Subsequently, the samples were maintained at 2 °C–8 °C and centrifuged at 3,000 rpm for 15 min. The resulting supernatant plasma was then collected, and the plasma melatonin concentration was determined using a human melatonin enzyme-linked immunosorbent assay kit (Wuhan Elabscience Biotechnology Co.,Ltd.).

After the experiment, the animals were euthanized by CO_2_ inhalation, with a CO_2_ displacement rate of 60% of the chamber volume per minute.

## 3 Results and discussion

### 3.1 Morphology of transdermal patch

An electret-permeation enhancer synergetic transdermal patch of melatonin with a size of 3 cm × 3 cm was prepared. The physical top view of the patch is shown in Figure 1a, with a flat and uniform appearance. Figure 1b shows the SEM image of the patch, which has a clear three-layer structure. The top is the protective layer composed of Glassine Paper, which plays a role in preventing drug-loading layer pollution and drug leakage. The middle is the drug-loading layer, which is mainly composed of polyacrylate PSA as a skeleton to load melatonin. Moreover, permeation enhancers and excipients are also added to PSA. In addition to storing and releasing drugs, it also used to bond the backing layer and skin. The bottom is the backing layer, a PP nonwoven electret, which provides mechanical support and protection, and meantime promotes the transdermal delivery of drugs by physical effect. The three layers are tightly bonded, forming a clear structure without gaps or bubbles.

**FIGURE 1:**
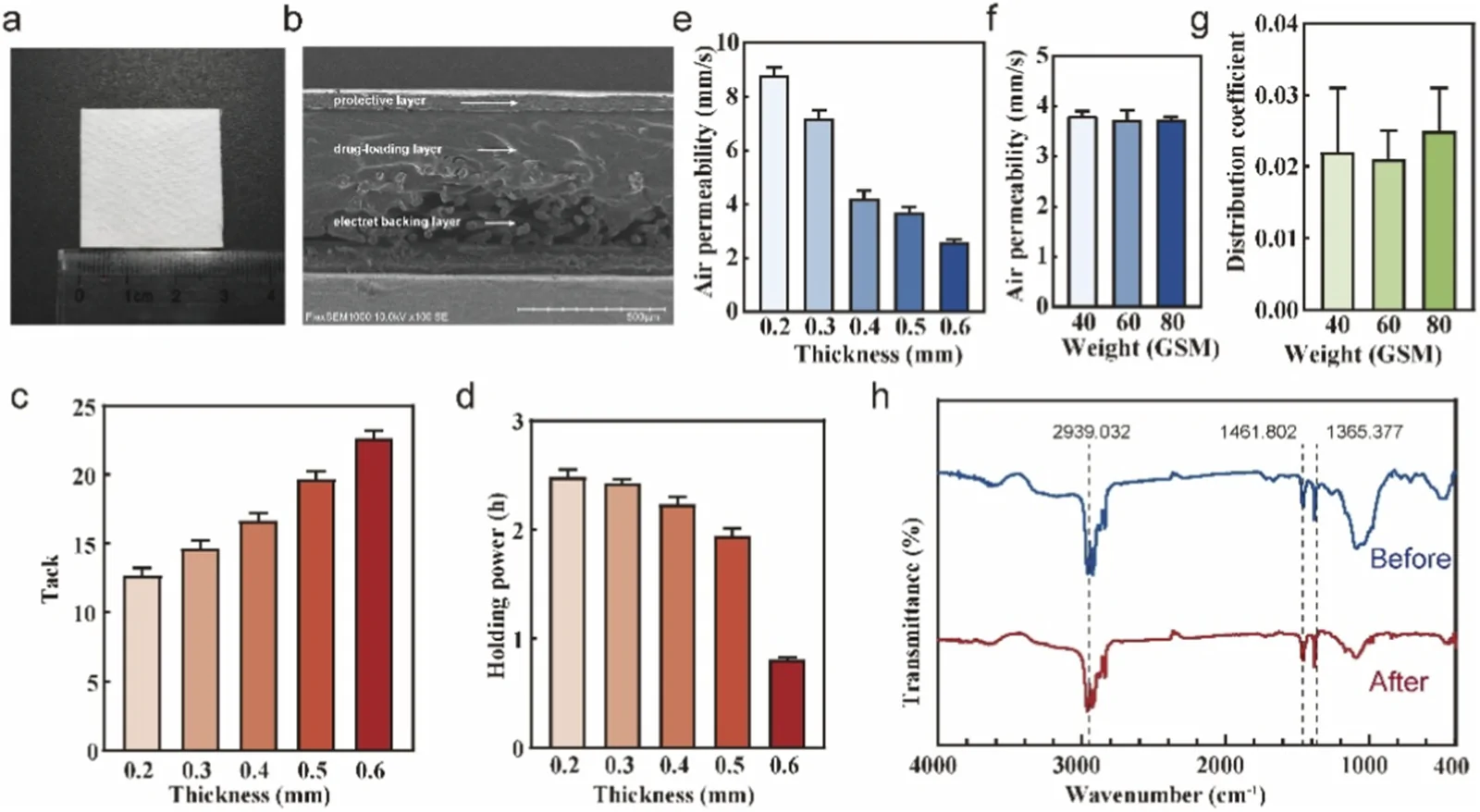
Characterization of melatonin transdermal patches: **(a)** photograph, **(b)** SEM image, **(c)** tack, **(d)** holding power **(e,f)** air permeability, **(g)** distribution coefficient, **(h)** FTIR spectra.

### 3.2 Characterization of drug-loading layer

The drug-loaded layer adheres the transdermal patch to the skin, required appropriate adhesion properties to ensure that the patch is fully and closely attached to the skin throughout the use and is easy to tear off after use (Bai et al., 2025). Generally, the adhesion with the skin can be reflected by the tack and the holding-power of the patch. Tack is the peeling resistance of the patch to the skin after the patch is bonded to the skin with a lighter pressure. Holding-power is the ability of the patch to resist creep damage caused by persistent shear force. The adhesion of the patches with different thickness of the drug-loaded layer is shown in Figures 1c,d. In a certain range, with the increase of the thickness of the drug-loaded layer, the tack of the patch increases while the holding-power of the patch decreases. Generally, the transdermal patch has appropriate tack when the maximum ball number of the test steel ball adhered to the transdermal patch is between 14 and 22. The holding power of the transdermal patch reflects the cohesion of PSA. For the patch required a long application time, the time to shed should be more than 1 hour. Therefore, the transdermal patch has appropriate adhesion when the thickness of the drug-loaded layer is 0.4–0.5 mm.

Air permeability is one of the most important performance parameters of transdermal patches, which determines the comfort of use. In general, high air permeability of the patch leads to dry skin and comfortable feeling. The air permeability of the patch refers to the air flow intensity per unit area under the action of constant pressure difference in unit time. The air permeability of the patches with different thickness of the drug-loaded layer is shown in Figure 1e. With the increase of the thickness of the drug-loaded layer, the closer the PSA structure is, the lower the air permeability is. When the thickness of the drug-loading layer was 0.4 mm and 0.5 mm, no significant difference was observed in the patch’s air permeability. To maximize the drug-loading capacity while maintaining optimal performance, a thickness of 0.5 mm was selected as the preferred parameter for the drug-loading layer.

### 3.3 Characterization of backing layer

For the backing layer, we studied the effect of the weight of PP nonwovens on the air permeability and distribution coefficient of the transdermal patch. The air permeability of PP nonwovens with different weight and thickness of 0.5 mm is shown in Figure 1f. In the range of 40–80 g/m^2^, there was no significant difference in the air permeability of the backing layer.

The distribution coefficient can characterize the drug absorption of the material. Higher distribution coefficient means stronger interaction between the material and the drug, indicating that the drug is more easily adsorbed by the material. For transdermal patches, the backing layer, as the outermost layer to protect and support the drug-loaded layer, needs to avoid the adsorption of drugs as much as possible. Low distribution coefficient of the backing layer indicates that the drug is stable in the drug-loaded layer and more easily released from the PSA to the human skin surface. As shown in Figure 1g, the distribution coefficients for melatonin of the three kinds of backing layers with different weight are all very small, indicating weak adsorption. Considering comprehensively, the backing layer is preferably 60 g/m^2^.

According to the molecular formula analysis of melatonin and PP, there is no reaction site between them. In order to verify that there is no new chemical bond between the drug and the backing layer, which leads to low drug release rate, the samples of 60 g/m^2^ backing layer before and after equilibrium in melatonin solution were tested by Fourier transform infrared spectroscopy, as shown in Figure 1h. The test results show that the characteristic peaks of the samples treated with melatonin solution are consistent with those of the blank samples. The absorption peaks at 2939.032 cm^-1^, 1,461.802 cm^-1^ and 1,365.377 cm^-1^ are all the stretching vibration of C-H in PP, and it is not observed the existence of characteristic peak of other new chemical bond or functional group such as benzene and N-H. These results demonstrate that melatonin does not chemically bind to PP fiber and is basically not adsorbed on it. Therefore, PP nonwoven has good chemical inertness and is suitable as a backing layer for transdermal patches.

### 3.4 Permeation-promoting effect of permeation enhancer

The chemical permeation enhancer (PE) is directly mixed into the PSA of the drug-loaded layer and is in direct contact with the skin when used. Common chemical permeation enhancers include terpenoids, ketones, fatty acids and other compounds. Three representative compounds were selected for subsequent research: menthol, which belongs to terpenoids, azone, which belongs to ketones, and oleic acid, which is a monounsaturated fatty acid. The optimal PE concentration typically ranges from 2% to 10% (w/w). Concentrations below this threshold exhibit insufficient enhancement efficacy, while excessive concentrations may induce cutaneous irritation. Therefore, three test concentrations (3%, 5%, and 10%) were systematically investigated.

Because part of the transdermal permeation of the drug is passive diffusion, that is, the drug diffuses from the high concentration side to the low concentration side. According to Fick’s law (Hashim et al., 2023), $\frac{dC}{dt}=-P\times \Delta C$ , where P is the permeability coefficient, $\frac{dC}{dt}$ is the change rate of the drug concentration in the blood, ΔC is the drug concentration gradient on both sides of the permeation. For a specific administration environment, the drug is rapidly diluted after entering the blood, the free drug concentration is low, and the outer melatonin content is continuously supplemented by the transdermal drug delivery system. Thus, ΔC can be seen as a constant. The higher the dissolution rate of the transdermal patch, the higher the concentration of the outer melatonin of the skin, the higher the ΔC , and the higher the transdermal rate of the drug. Therefore, the *in vitro* release of melatonin in PSA was first tested, as shown in Figures 2a–c. With the increase of dissolution time, the dissolution rate of melatonin decreased. The release rate of melatonin in PSA was 20%–42% at 1 h and 62%–89% at 8 h. Three kinds of PE could increase the release rate of melatonin from PSA, and the higher the concentration of PE, the stronger the promotion effect. This is because the higher the concentration of PE, the larger the molecular gap in PSA, and the easier it is for melatonin to escape through the pores. Among them, oleic acid has the strongest promoting effect, which is due to the unsaturated long-chain structure of oleic acid, which is easy to cause large molecular gaps in PSA.

**FIGURE 2:**
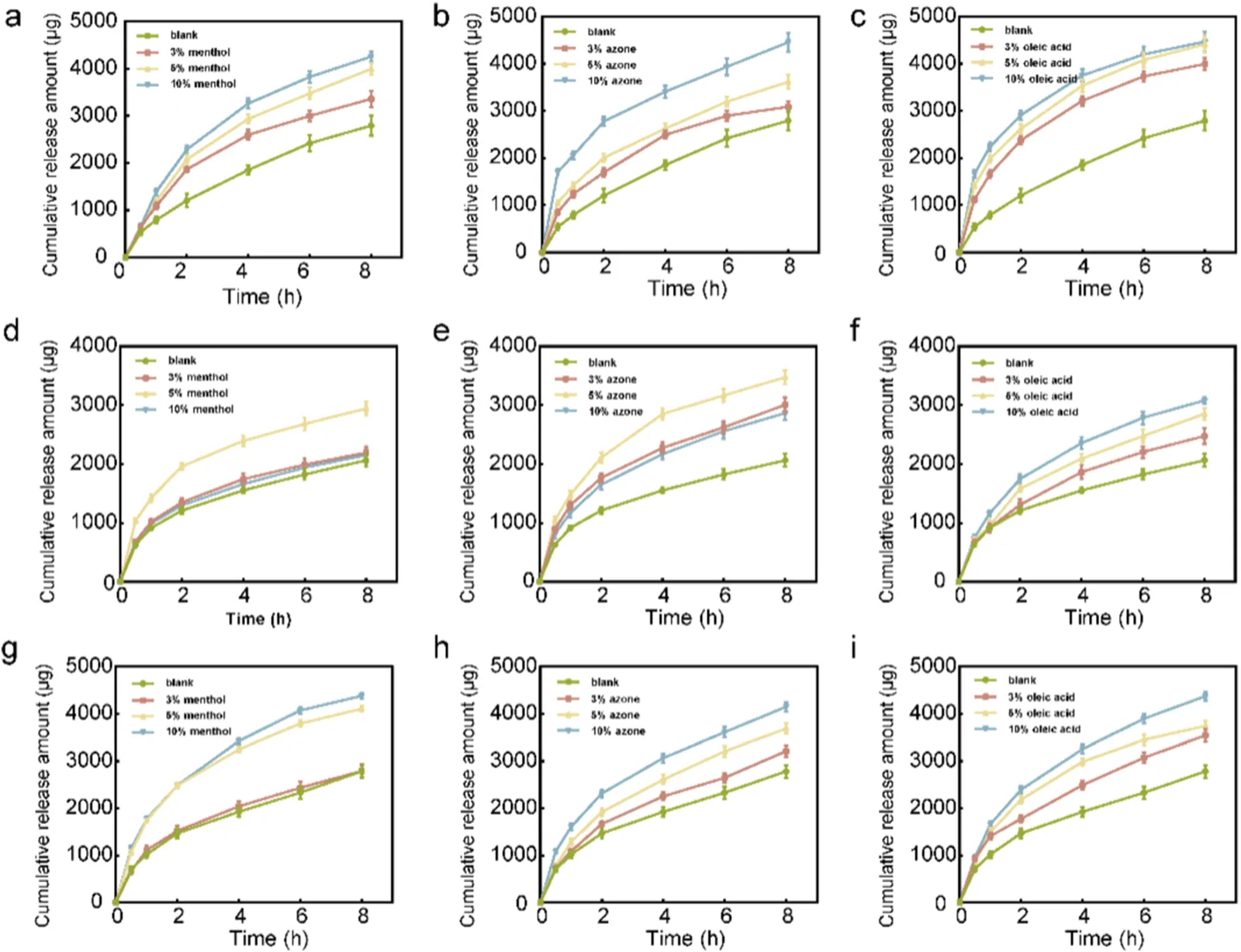
Effects of different permeation enhancer formulations on the performance of transdermal patches: **(a–c)** *in vitro* release properties of melatonin in patches, **(d–f)** *in vitro* transdermal permeation properties (intact skin), **(g–i)** *in vitro* transdermal permeation properties (viable skin).

We then systematically evaluated the effects of various PEs at different concentrations on melatonin’s *in vitro* transdermal permeation. For intact skin, the 8-h cumulative permeation of melatonin ranged from 42% to 71%, with distinct concentration-dependent patterns observed: menthol and azone both showed optimal enhancement at 5% concentration (5% > 3% ≈ 10%), while oleic acid exhibited a strictly dose-dependent increase (10% > 5% > 3%) (Figures 2d–f). In comparison, due to the absence of the stratum corneum barrier, viable skin demonstrated significantly enhanced permeation (57%–89% at 8 h), where all three PEs displayed clear positive concentration-response relationships, with permeation efficiency progressively increasing at higher concentrations (Figures 2g–i).

The three PEs exhibit distinct mechanisms of action, leading to differential effects on melatonin transdermal permeation and optimal concentration thresholds. As a terpene compound, menthol preferentially partitions into and inserts itself within the intercellular lipids of the stratum corneum, where it reversibly disrupts the highly ordered lipid bilayer structure and increases its fluidity. This action creates drug permeation pathways in the skin, thereby significantly enhancing the transdermal delivery of drugs. Consequently, within a certain concentration range, the permeation-enhancing effect increases with rising menthol concentration. Conversely, excessively high concentrations of menthol may over-disrupt the lipid architecture, leading to a chaotic disruption of the stratum corneum barrier function. This renders the drug diffusion pathway tortuous and unfavorable, ultimately diminishing the permeation-enhancing effect. In this study, 5% was identified as the optimal concentration for the permeation-enhancing effect of azone. At this concentration, azone interacts with intercellular or biomembrane lipids to create transient pores through partial lipid dissolution. Suboptimal concentrations fail to establish effective pores, whereas excessive concentrations induce stratum corneum over-hydration through azone’s hydration mechanism, resulting in melatonin molecule entrapment. Oleic acid uniquely enhances melatonin diffusion across both stratum corneum and viable skin by intercalating into the hydrophobic domains of lipid bilayers. Its cis-configuration long-chain structure induces phospholipid membrane fluidization and torsion, generating permeable microchannels. Based on comprehensive permeation enhancement assessment, the optimized chemical parameters were determined as 5% menthol, 5% azone, and 10% oleic acid.

### 3.5 Permeation-promoting effect of electret

The skin functions as a biological electret capable of sustaining long-term charge storage and maintaining a polarized state. Alterations in the skin’s electret state can induce not only modifications in the electrical architecture of tissues, cells, and molecules, but also conformational changes at the molecular level, which may facilitate enhanced transdermal drug permeation. Exogenous electrets serve as passive ion-driving sources that deliver sustained electrostatic fields and microcurrents to the skin, thereby modulating its electret state and promoting drug absorption (Jiang et al., 2012; Jiang et al., 2012). The underlying mechanisms involve: (1) High-voltage electret application generates a significant transmembrane potential difference, inducing molecular reorientation of the intercellular lipid bilayers within keratinocytes to create transient permeable channels; (2) The surface potential of the electret sustains channel patency post-formation; (3) During transdermal transport, the microcurrent generated by detrapped charges enhances aqueous phase fluidity, consequently augmenting drug permeation.

The effects of positive and negative electrets with varying field strengths on melatonin’s *in vitro* release and transdermal permeation were systematically evaluated. While electrets showed no significant influence on melatonin’s *in vitro* release profile (Figures 3a,b), both polarities significantly enhanced melatonin diffusion through both stratum corneum and viable skin layers, with positive electrets achieving 16.72%–58.68% permeation enhancement and negative electrets demonstrating superior efficacy (48.71%–101.77% increase). As illustrated in Figures 3c,d, intact skin exhibited two characteristic responses: (1) a direct correlation between electret field strength and permeation enhancement within each polarity group, and (2) consistently greater enhancement by negative *versus* positive electrets at equivalent field strengths, a phenomenon attributable to the inherent positive charge of human skin surface which facilitates stronger microcurrent generation by negative-potential electrets. Notably, viable skin followed similar trends (Figures 3e,f), though the absolute permeation increase appeared proportionally smaller due to higher flux resulting from stratum corneum absence. Consequently, negative-charged electrets were identified as the optimal physical enhancers for melatonin transdermal delivery, with three graded-strength negative electrets selected as preferred parameters through comprehensive evaluation.

**FIGURE 3:**
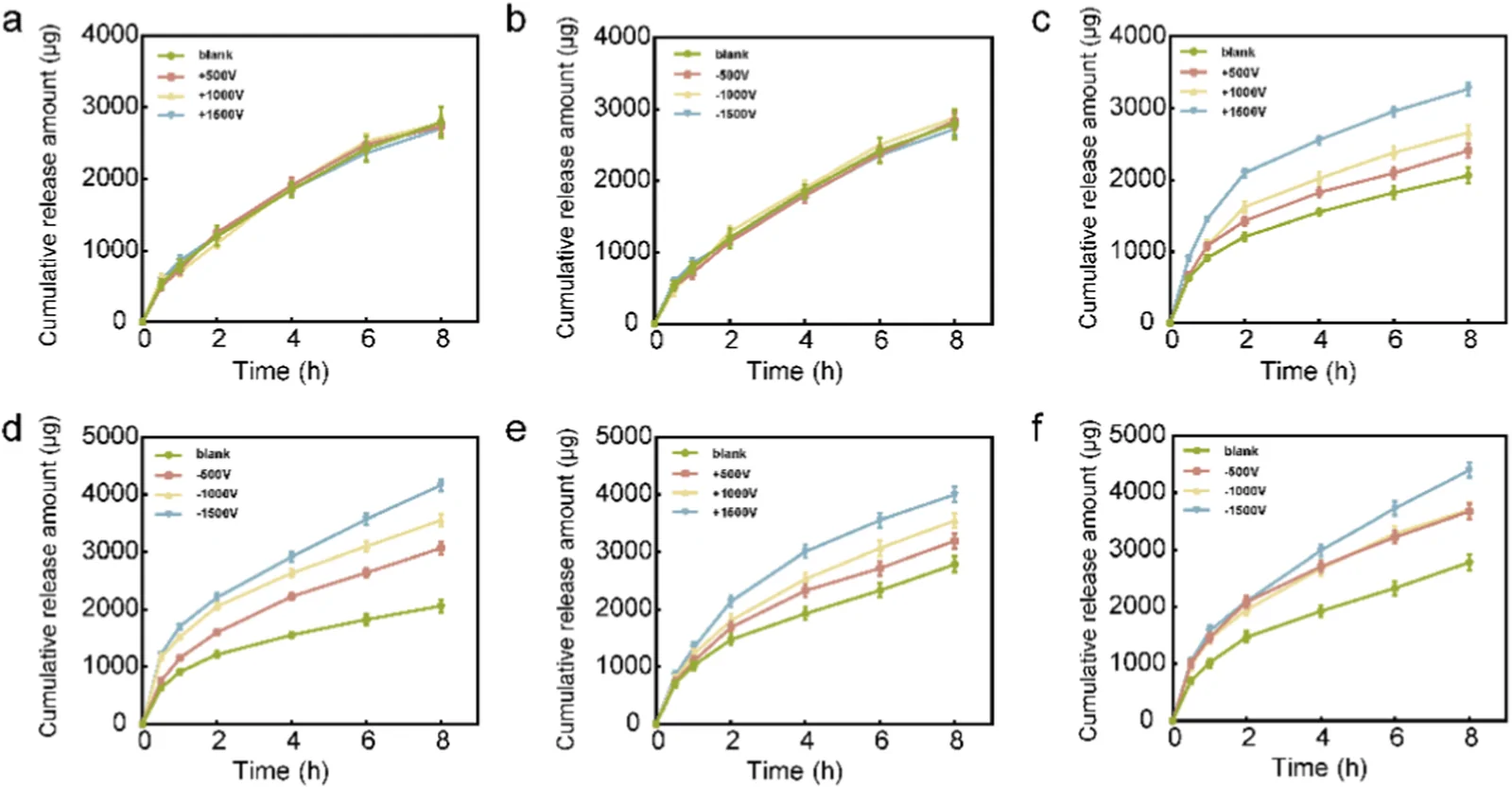
Effects of different electret parameters on the performance of transdermal patches: **(a,b)** *in vitro* release properties of melatonin in patches, **(c,d)** *in vitro* transdermal permeation properties (intact skin), **(e,f)** *in vitro* transdermal permeation properties (viable skin).

Ambient humidity is known to be a critical factor influencing the charge storage stability of electret materials. We measured the decay of surface potential over time for +1000 V electrets at 37 °C under ambient humidity conditions of 30%, 45%, and 60%, respectively (Supplementary Figure S1). The results showed that under different ambient humidity conditions, the surface potential of the electrets remained relatively stable within 36 h (decay rate <10%), and the decay rate within 12 h (the conventional usage time) was less than 6%, demonstrating that the electrets possess sufficient charge storage longevity during application.

### 3.6 Permeation-promoting effect of electret-permeation enhancer synergetic transdermal patch

The principle of physical permeation and chemical permeation is different. Usually, they can work on the skin surface at the same time. When the parameters are appropriate, they promote each other to achieve the effect of synergistic permeation. As shown in Figure 4, transdermal permeation behavior of patches with different parameters was evaluated *in vitro* to screen the parameter set that yielded the optimal synergistic effect for subsequent experiments. For intact skin, there is a synergistic effect between the permeation enhancer and the electret, and the permeation efficiency and total transdermal diffusion of melatonin are better than the single effect. For menthol, the synergistic effect of −500 V electret and −1000 V electret is weak. It may be because when 5% concentration of menthol acts on the skin, it increases the water content of the stratum corneum of the skin. When the potential difference is not high, the electrostatic field is difficult to play a role in the environment with high water content, so the synergistic effect is not obvious. The electret with high potential difference carries more charges, and the electric field environment formed destroys the lipid structure of the stratum corneum. Menthol further acts on the intercellular substance, prompting the increase of fissures between epidermal cells, thereby reducing the skin's blocking effect on drugs. Azone dissolves intercellular lipids and forms a gap, while the electret also destroys the lipid structure, and the two effects are easy to interfere. Therefore, when azone is combined with the electret, the synergistic effect is not good. The synergistic effect of oleic acid and electret is the best, and the total amount of transdermal diffusion is the most obvious. This is because both of them work on the lipid structure, but act in different directions. Oleic acid macromolecules are inserted into the lipid bilayer to reverse the pathway, and the electrostatic field disrupts the tight structure of the lipid, which can act on the stratum corneum cells that are not affected by oleic acid. Therefore, the synergistic effect of 10% oleic acid and electret can maximize the transdermal permeation and absorption of melatonin compared with other prescriptions. The synergistic effect of azone and electret mainly improves the permeation efficiency of viable skin, and the permeation effect on the stratum corneum is not obvious. This is because the permeation effect of both is on the viable skin, and the effect on the skin surface with strong stratum corneum barrier is not good. The synergy of menthol with oleic acid and electret is mainly to promote the permeation of melatonin in the stratum corneum, so the transdermal diffusion efficiency in viable skin and the transdermal diffusion efficiency in intact skin are less improved. According to the above, the preferred synergistic prescription was 10% oleic acid and-1500 V electret. The optimized patch showed an *in vitro* cumulative transdermal permeation rate of 77.06%.

**FIGURE 4:**
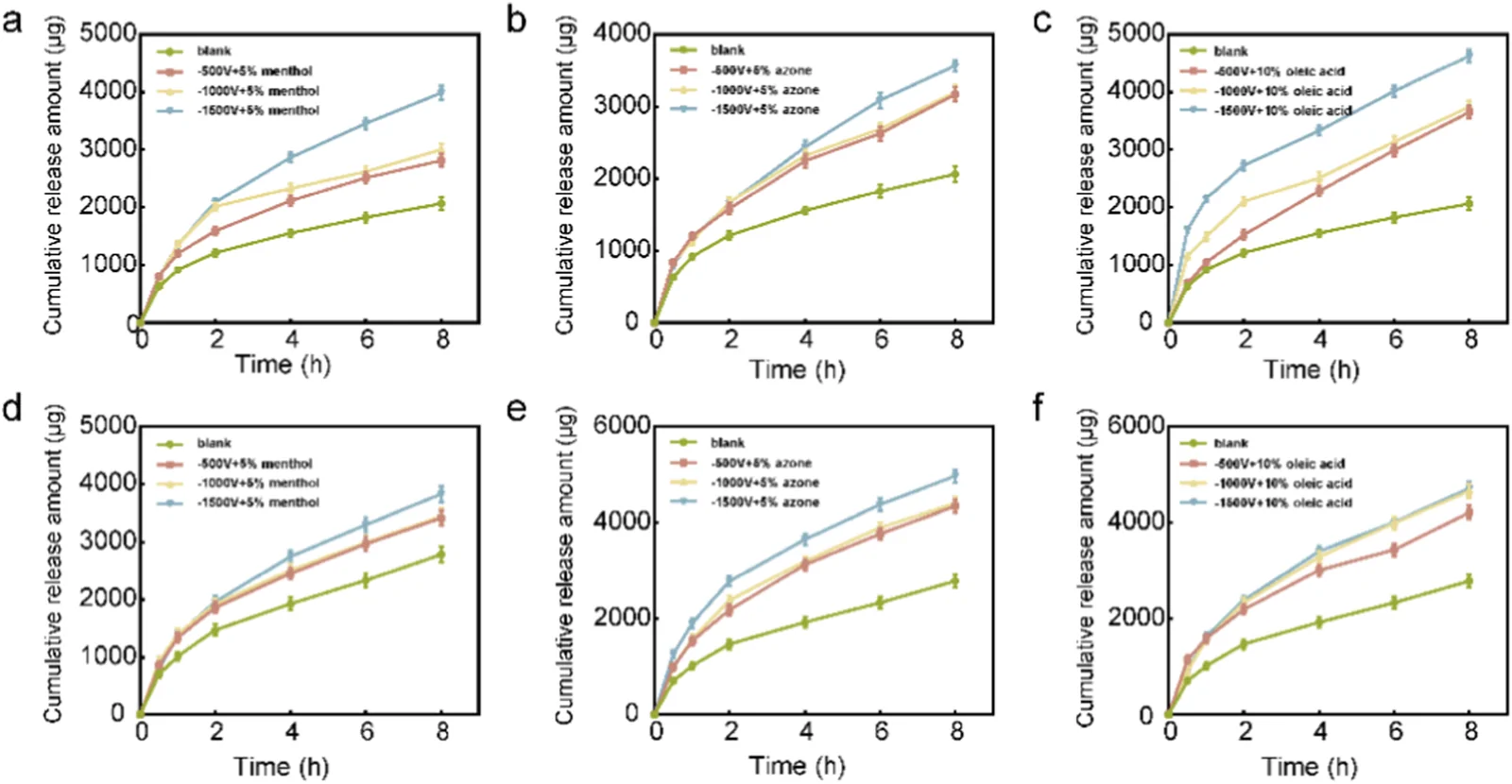
Effects of different formulations on the *in vitro* transdermal permeation properties of patches: **(a–c)** intact skin, **(d–f)** viable skin.

The *in vitro* release behavior of the transdermal patch was fitted by four models: zero-order equation, first-order equation, Higuchi model and Ritger-peppas model. The fitting results are shown in Table 1. The results show that the correlation degree of the four fitting methods is different. Among them, the correlation coefficient of Higuchi fitting is the highest, which is 0.99357. The Higuchi model (Ameen et al., 2019) is the relationship model between drug release and time in homogeneous skeleton type and heterogeneous multi-channel skeleton type solid preparations. The cumulative drug release exhibits a linear relationship with the square root of time (t^(1/2)), which is characteristic of drug release kinetics from solid matrix dosage forms. This principle is widely applied in calculating drug release profiles for sustained-release preparations, microspheres, and similar delivery systems. Higuchi model fitting further demonstrated that melatonin in the prepared transdermal delivery system was homogeneously dispersed and dissolved within the polyacrylate PSA matrix, with drug release occurring through skin pores. Transdermal diffusion studies on skin models showed optimal consistency with the Higuchi model, yielding a high correlation coefficient.

**TABLE 1:** Fitting equations of *in vitro* drug release models.

Release kinetic models	Fitting equation	Correlation coefficient *R* ^2^
Zero-order fitting	$f\left(x\right)=377.19734x+1724.23517$	0.96528
First-order fitting	$f\left(x\right)=4177.03032\left(1-e^{-0.62381x}\right)$	0.83981
Higuchi fitting	$f\left(x\right)=1360.75484x^{\frac{1}{2}}+717.39023$	0.99357
Ritger-peppas fitting	$f\left(x\right)=2080.99054x^{0.37258}$	0.99155

### 3.7 Biocompatibility of electret-permeation enhancer synergetic transdermal patch

The biocompatibility of the transdermal patch was evaluated through sensitization tests in rats and cytotoxicity tests. The dorsal hair of rats was removed, and the patch with optimized parameters was applied. Upon removal at 10 h, the application site was inspected. No signs of erythema or edema were observed (Figures 5a–c), indicating the absence of dermal irritation or allergic reaction, which supports its good biocompatibility. As shown in Figure 5d, compared to the blank culture plate, the cell viability of the drug-loaded control patch group was 94.25% at 24 h, while that of the drug-loaded transdermal patch group was 91.00%. The live/dead cell staining images also demonstrated the good biocompatibility of the patches (Figure 5e), suggesting their potential for clinical application.

**FIGURE 5:**
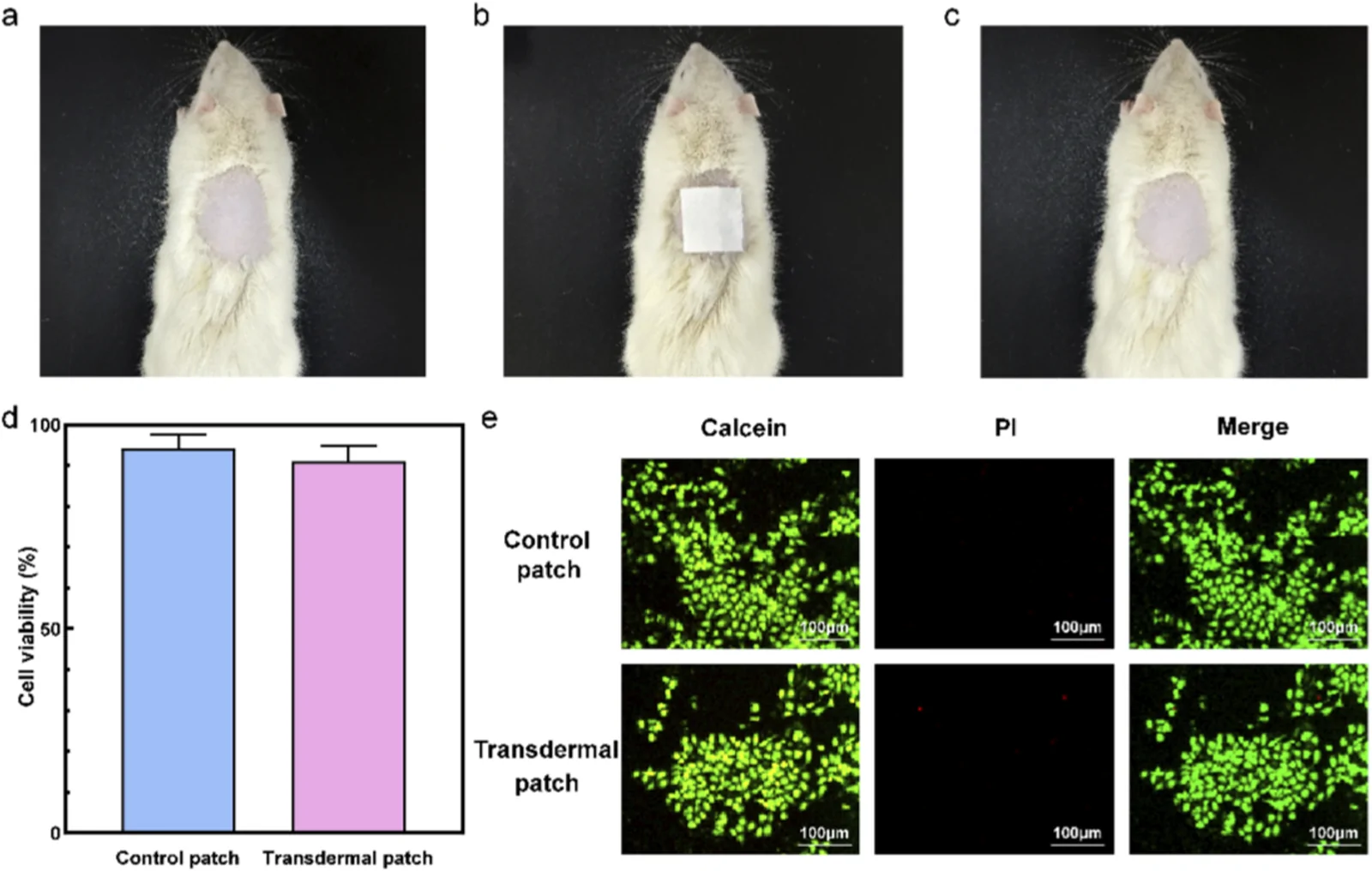
Biocompatibility of patches: **(a–c)** sensitisation test of transdermal patch in SD rats skin, **(d)** cell viability using patch leachate by CCK-8 kit and **(e)** images of live-dead staining after 24 h (scale bar 100 μm).

### 3.8 *In vivo* pharmacokinetic analysis of electret-permeation enhancer synergetic transdermal patch

SD rats with insomnia were randomly divided into three groups: a control group, a drug-loaded control patch group, and a drug-loaded transdermal patch group, with three rats in each group. They were respectively given no treatment, application of the drug-loaded control patch, and application of the drug-loaded transdermal patch, and were observed in the meantime. Before treatment, the rats in all three groups were in an alert state. After treatment, the rats in the control group and the drug-loaded control patch group remained excited, while the rats in the drug-loaded transdermal patch group fell asleep after 20–30 min.

Subsequently, an *in vivo* pharmacokinetic analysis of melatonin was performed on the three groups (Figure 6). The results showed that in the drug-loaded control patch group, a small amount of melatonin slowly permeated the skin and entered the systemic circulation after patch application, which is attributed to the relatively low molecular weight and lipophilicity of melatonin, coupled with the stratum corneum hydration induced by the patch. In contrast, in the drug-loaded transdermal patch group, under the synergistic enhancing effects of the electret and permeation enhancer, melatonin penetrated the skin relatively rapidly and extensively, entering the systemic circulation. This led to a significant increase in plasma melatonin levels within a short period, facilitating the rapid induction of sleep in the rats. After reaching the peak concentration, the plasma melatonin level declined gently and maintained an effective concentration (over 5 ng/mL) for up to 8 h, which contributed to the maintenance of sleep and improvement of sleep quality in the rats. During application, the maximum plasma concentration of melatonin exceeded 30 ng/mL, peaking at 1 h. Due to the sustained-release characteristics of the transdermal patch and the continuous transdermal transport of melatonin, melatonin is gradually delivered into the systemic circulation, thereby counterbalancing its metabolic clearance. This results in relatively stable plasma melatonin levels without a sharp decline, maintaining a high concentration for up to 8 h. Such a pharmacokinetic profile meets the requirements for circadian rhythm regulation, partially mimics physiological melatonin release patterns, and may offer potential efficacy in circadian rhythm regulation.

**FIGURE 6:**
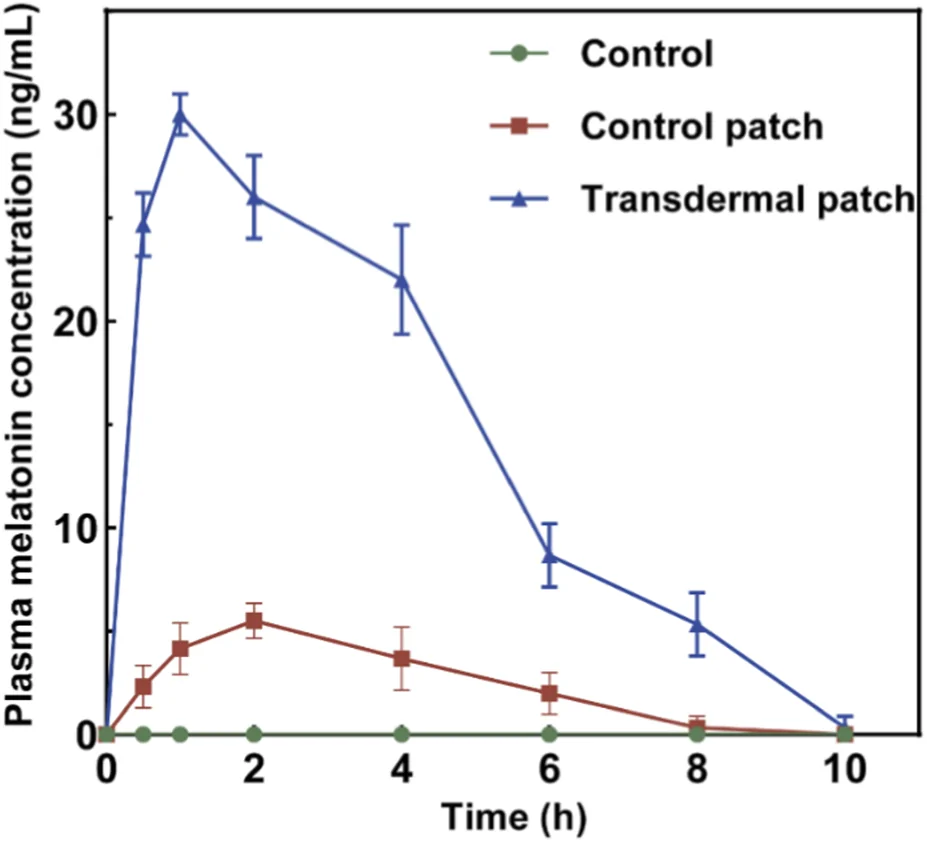
*In vivo* experiment with transdermal patch: plasma melatonin concentration of SD rats within 10 h.

## 4 Discussion

Circadian rhythm disruption affects up to one-third of the adult population worldwide (Perlis et al., 2022). Melatonin, a critical neuroendocrine hormone, can effectively address such disturbances. Moreover, with a short half-life of merely half an hour and an absence of hangover-like side effects, it presents a favorable safety profile for therapeutic use (Amstrup et al., 2015). Compared with oral administration, transdermal delivery of melatonin has many advantages, such as high bioavailability, stable plasma concentration, and small hepatorenal toxicity.

For transdermal delivery of melatonin, Qi et al. (2021) developed a Silk Fibroin Microneedle Patch for melatonin transdermal delivery patches, which reached a maximum plasma concentration of about 10 ng/mL at 0.31 h in rats and maintained a plasma concentration of more than 5 ng/mL for 4–6 h. Li et al. (2025) improved the previous study. The microneedle patch reached the maximum plasma concentration of 25–30 ng/mL at 1 h in rats, and maintained the plasma concentration of more than 5 ng/mL for 8 h. As a transdermal delivery of melatonin, microneedles have some disadvantages, such as small drug loading and certain destructiveness to the skin. In this study, an electret-permeation enhancer synergetic textile transdermal patch of melatonin was prepared. The synergistic effect of electret and permeation enhancer on stratum corneum lipid molecules was used to expand the skin channel and increase the transdermal efficiency of melatonin. Since the drug is directly dispersed in the PSA, the drug loading of the transdermal patch has a wide range, which is conducive to the improvement of blood drug concentration. The synergistic effect of electret and permeation enhancer makes the transdermal delivery of melatonin more efficient and lasting, and has no damage to the skin. In addition, different electrets and permeation enhancers lead to different *in vitro* transdermal effects. Transdermal patches can achieve different blood concentration curves by adjusting the preparation parameters to meet different needs and have great adjustability. *In vivo* studies demonstrated that the application of the optimized transdermal patch enabled rapid sleep onset in insomniac rats and maintained an elevated plasma concentration (exceeding 5 ng/mL) for up to 8 h, with a peak concentration surpassing 30 ng/mL achieved within 1 h. These results indicate the significant potential of the textile transdermal patch for circadian rhythm regulation application.

It should be noted that the present study has not yet conducted *in situ* spectroscopic or thermal characterizations of stratum corneum lipid ultrastructure, such as ATR-FTIR or differential scanning calorimetry (DSC). Nevertheless, based on two well-established independent mechanisms, namely, oleic acid-induced lipid disordering (Mao et al., 2013; Jiang et al., 2003) and electret-induced lipid structural transitions from gel to liquid crystal (Cui et al., 2011), we logically deduce that the complementary perturbation of both agents at the same barrier site constitutes the most reasonable molecular basis for explaining the observed synergistic penetration-enhancing effect. Direct experimental verification of this deduction will be a key focus of subsequent investigations.

For further clinical translation of the patch, subsequent studies should comprehensively evaluate its stability during actual use (including the effects of mechanical deformation and ambient humidity on electret charge stability), transdermal efficacy, skin barrier recovery function after long-term repeated administration, and potential systemic effects through animal experiments and human wearing tests. However, the preliminary data have already demonstrated the feasibility of this strategy as a clinically viable option. In addition, the textile-based electret backing layer used in this study is based on the well-established corona charging process for melt-blown polypropylene nonwoven fabric, which has been widely applied in industrial production; the drug loading into the pressure-sensitive adhesive is performed by a coating process that is compatible with existing transdermal patch production lines. This also indicates the scalability of the patch system manufacturing.

## 5 Conclusion

Based on a physicochemical synergistic permeation -enhancement strategy, this study successfully developed a melatonin textile transdermal patch leveraging the cooperative action of electret and permeation enhancer, effectively addressing the challenge of balancing efficiency and safety in existing transdermal delivery systems. The patch adopts a three-layer composite structure: a textile-based electret as the backing layer, a pressure-sensitive adhesive containing melatonin and a permeation enhancer as the drug-loaded layer, and a leak-proof protective layer. We optimized the patch’s permeability, adhesion, and distribution coefficient, and investigated the effects of electrets with different polarities and strengths, as well as various types and concentrations of permeation enhancers, on the transdermal permeation of melatonin *in vitro*. The results demonstrated that the combination of a −1500 V electret and 10% oleic acid exhibited the highest synergistic permeation enhancement efficiency. *In vivo* pharmacokinetic experiments further validated the clinical potential of the patch. In an insomniac rat model, the optimized transdermal patch enabled effective and sustained melatonin delivery, maintaining high plasma levels for up to 8 h, with a peak concentration exceeding 30 ng/mL at 1 h. The transdermal patch efficiently and persistently promoted melatonin delivery and allowed flexible customization of the drug’s transdermal profile through different combinations. In summary, this study not only realizes an efficient, long-acting, tunable, and non-invasive textile-based platform for transdermal melatonin delivery but also proposes a physicochemical synergistic permeation-enhancement strategy. This strategy can be extended to the transdermal delivery of other neuroendocrine small-molecule hormones, offering broad prospects for developing safe, efficient, and readily scalable textile-based transdermal drug delivery systems.

## Data Availability

The original contributions presented in the study are included in the article/Supplementary Material, further inquiries can be directed to the corresponding author.
