# Supplementary material for: A melatonin textile transdermal patch based on physicochemical synergistic permeation-enhancement strategy for circadian rhythm regulation
Source: Front Bioeng Biotechnol. 2026 Oct 8;14:1913854. doi: 10.3389/fbioe.2026.1913854 (PMC13687086; doi:10.3389/fbioe.2026.1913854)
Supplement: Supplementary file 1 [file Supplementaryfile1.docx]

**Fig. S1** Surface potential variations of electret under different ambient humidity (RH) conditions.
